# Supplementary material for: Hyperglycemia compromises Rat Cortical Bone by Increasing Osteocyte Lacunar Density and Decreasing Vascular Canal Volume
Source: Commun Biol. 2020 Jan 9;3:20. doi: 10.1038/s42003-019-0747-1 (PMC6952406; doi:10.1038/s42003-019-0747-1)
Supplement: Supplementary file 1 — Description of Additional Supplementary Files [file 42003_2019_747_MOESM1_ESM.pdf]

Description of Additional Supplementary Files

File Name: **Supplementary Data 1**
Description: **Source data**
