# Supplementary material for: Hyperglycemia compromises Rat Cortical Bone by Increasing Osteocyte Lacunar Density and Decreasing Vascular Canal Volume
Source: Commun Biol. 2020 Jan 9;3:20. doi: 10.1038/s42003-019-0747-1 (PMC6952406; doi:10.1038/s42003-019-0747-1)
Supplement: Supplementary file 3 — Reporting Summary [file 42003_2019_747_MOESM3_ESM.pdf]

nature research

Corresponding author(s): John E. Davies

Last updated by author(s): Dec 4, 2019

Reporting Summary

Nature Research wishes to improve the reproducibility of the work that we publish. This form provides structure for consistency and transparency in reporting. For further information on Nature Research policies, see Authors & Referees and the Editorial Policy Checklist.

Statistics

For all statistical analyses, confirm that the following items are present in the figure legend, table legend, main text, or Methods section.

n/a	Confirmed	
☐	☒	The exact sample size (n) for each experimental group/condition, given as a discrete number and unit of measurement
☐	☒	A statement on whether measurements were taken from distinct samples or whether the same sample was measured repeatedly
☐	☒	The statistical test(s) used AND whether they are one- or two-sided<br>*Only common tests should be described solely by name; describe more complex techniques in the Methods section.*
☒	☐	A description of all covariates tested
☐	☒	A description of any assumptions or corrections, such as tests of normality and adjustment for multiple comparisons
☐	☒	A full description of the statistical parameters including central tendency (e.g. means) or other basic estimates (e.g. regression coefficient) AND variation (e.g. standard deviation) or associated estimates of uncertainty (e.g. confidence intervals)
☐	☒	For null hypothesis testing, the test statistic (e.g. F, t, r) with confidence intervals, effect sizes, degrees of freedom and P value noted<br>*Give P values as exact values whenever suitable.*
☒	☐	For Bayesian analysis, information on the choice of priors and Markov chain Monte Carlo settings
☒	☐	For hierarchical and complex designs, identification of the appropriate level for tests and full reporting of outcomes
☒	☐	Estimates of effect sizes (e.g. Cohen's d, Pearson's r), indicating how they were calculated

Our web collection on statistics for biologists contains articles on many of the points above.

Software and code

Policy information about availability of computer code

Data collection	Amira 6.3 (ThermoFisher Scientific, USA), CT Analyzer 1.16.4.1 (Bruker, Belgium),
Data analysis	GraphPad Prism 6 (GraphPad Software Inc., USA), R studio 3.4.0

For manuscripts utilizing custom algorithms or software that are central to the research but not yet described in published literature, software must be made available to editors/reviewers. We strongly encourage code deposition in a community repository (e.g. GitHub). See the Nature Research guidelines for submitting code & software for further information.

Data

Policy information about availability of data

All manuscripts must include a data availability statement. This statement should provide the following information, where applicable:

- Accession codes, unique identifiers, or web links for publicly available datasets
- A list of figures that have associated raw data
- A description of any restrictions on data availability

The majority of the raw data were generated at the Canadian Light Source large-scale facility. Source data for figures are provided with the paper as supplementary data.

Field-specific reporting

Please select the one below that is the best fit for your research. If you are not sure, read the appropriate sections before making your selection.

☒ Life sciences ☐ Behavioural & social sciences ☐ Ecological, evolutionary & environmental sciences

Life sciences study design

All studies must disclose on these points even when the disclosure is negative.

Sample size	Since the requested beam time in our proposal to use synchrotron radiation micro-CT at Canadian Light Source, SK, Canada had to be a reasonable time, we decided our sample size based on the time limitation.
Data exclusions	No data were excluded
Replication	We used an STZ-induced hyperglycemic rat model, which is a commonly used animal model, and we stated the blood glucose limit of animals which are considered as hyperglycemic (blood glucose>15 mmol/L). We used commercially available analysis tools (AMIRA, CTAn) to analyze our Micro-CT data. Therefore, we believe our results will be reproducible when the defined methods have been followed.
Randomization	Same strain and gender rats (male Wistar Albino) has been randomly chosen to receive STZ injections to induce hyperglycemia.
Blinding	Only the sample numbers have been used during the data collection.

Reporting for specific materials, systems and methods

We require information from authors about some types of materials, experimental systems and methods used in many studies. Here, indicate whether each material, system or method listed is relevant to your study. If you are not sure if a list item applies to your research, read the appropriate section before selecting a response.

Materials & experimental systems

n/a	Involved in the study	
☒	☐	Antibodies
☒	☐	Eukaryotic cell lines
☒	☐	Palaeontology
☐	☒	Animals and other organisms
☒	☐	Human research participants
☒	☐	Clinical data

Methods

n/a	Involved in the study	
☒	☐	ChIP-seq
☒	☐	Flow cytometry
☒	☐	MRI-based neuroimaging

Animals and other organisms

Policy information about studies involving animals; ARRIVE guidelines recommended for reporting animal research

Laboratory animals	Rat, Wistar Albino, Male, 11-week old.
Wild animals	The study did not involve wild animals.
Field-collected samples	The study did not involve samples collected from the field.
Ethics oversight	All experimental protocols were approved by the Ethics Committee of Animal Research at the University of Toronto (Protocol # 20011729).

Note that full information on the approval of the study protocol must also be provided in the manuscript.
